# Determinants of Adherence to Antiretroviral Therapy among HIV-Infected Patients in Africa

**DOI:** 10.1155/2012/574656

**Published:** 2012-02-22

**Authors:** Ayalu A. Reda, Sibhatu Biadgilign

**Affiliations:** ^1^Department of Public Health, College of Health Sciences, Haramaya University, P.O. Box 1014, Harar, Ethiopia; ^2^Department of Epidemiology and Biostatistics, Jimma University, P.O. Box 24414, Jimma, Ethiopia

## Abstract

*Background*. There are only a few comprehensive studies of adherence to ART and its challenges in Africa. This paper aims to assess the evidence on the challenges and prospects of ART adherence in sub-Saharan Africa. *Methods*. The authors reviewed original and review articles involving HIV-positive individuals that measured adherence to ART and its predictors in the past decade. *Findings*. Against expectations, sub-Saharan Africa patients have similar or higher adherence levels compared to those of developed countries. The challenges to ART adherence include factors related to patients and their families, socioeconomic factors, medication, and healthcare systems. *Conclusion*. Despite good adherence and program-related findings, antiretroviral treatment is challenged by a range of hierarchical and interrelated factors. There is substantial room for improvement of ART programs in sub-Sahara African countries.

## 1. Introduction

Acquired immune deficiency syndrome (AIDS) is one of the most destructive epidemics the world has ever witnessed. Presently an estimated 33.4 million people are living with HIV worldwide, nearly two-thirds of these live in sub-Saharan Africa [1]. 

Antiretroviral therapy (ART) has shown to delay progression to AIDS, resulting in a greater and more sustained virologic and immunologic response [2] and improve survival [3]. In sub-Saharan Africa, there has been a dramatic increase in the number of HIV/AIDS patients on antiretroviral treatment from just 100,000 persons in 2003 to 3.9 million in 2009 involving close to 40% of those in need of the treatment [4]. Two sub-Saharan Africa countries, Botswana and Rwanda, have achieved universal access target (treatment coverage of 80% or more of patients in need) at the end of 2009 [4], while countries such as Ethiopia, Zambia, Namibia, and Senegal are moving closer to the same target having covered 50–80% of patients in need of treatment [4].

According to recent studies, ART regimens require 70–90% adherence in order to be effective [5]. However, sustaining adherence to antiretroviral therapy (ART) over the long term requires accurate and consistent monitoring, and this is a particular challenge for countries in sub-Saharan Africa [5]. It is further challenged by various social and clinical obstacles [5] where inadequate suppression of viral replication by ART are resulting due to poor adherence to therapy, low potency of the antiretroviral regimens, viral resistance to antiretroviral medications, and pharmacokinetic interactions [6] causing inadequate drug delivery [5, 7]. The transmissibility of the antiretroviral resistant viruses from person to person further compounds the problem as a clinical and public health challenge [8, 9].

Adherence is defined as taking medications or interventions correctly according to prescription. There are different methods for assessing adherence and the level of adherence is specific not only to places and patient groups but also to the method of adherence measurement used [10]. They include direct methods such as biologic markers and body fluid assays, or indirect methods such as self-report, interview, pill counts, pharmacy records, computerized medication caps, and viral load monitoring. While a combination of these methods may be employed, patient self-report is the most widely used [11] given its ease of implementation and use of already existing resources. Studies have also indicated that self-reports correlate well with both viral load and clinical outcomes [12, 13]. Use of computerized medication caps and monitoring of surrogate markers seems reliable and less prone to respondent bias. However, the advanced technology, high cost, and logistic requirements have precluded their wider application in sub-Saharan Africa [14]. In developing countries, pharmacy refill reports and self-reports are commonly implemented for adults [5, 15], while caregiver reports are employed for children [11, 16, 17]. Currently, there are no gold standard methods for measuring adherence [5].

There are only very few studies that investigate adherence to ART in sub-Saharan Africa. The aim of this paper is to assess the challenges of adherence to ART and to identify the factors that contribute to poor adherence. 

### 1.1. Current Estimates of Adherence

Studies indicate that despite earlier fears of poor medication adherence [6, 18], patients in developing countries are able to achieve adherence levels similar to or higher than those of patients in developed countries [19]. For instance, a review by Vreeman and colleagues indicated that the majority of the studies in developing countries report adherence levels of more than 75% (range 45–100%) [11], while in developed countries the majority report less than 75% (range 20–100%) [17]. Another systematic review by Mills and colleagues obtained a pooled estimate of adequate adherence by sub-Saharan Africa patients of 77% (95% confidence interval, 68–85%; based on a total of 12,116 patients), whereas the figure for North American patients was 55% (95% confidence interval 49–62%; based on a total of 17,573 patients) [20]. The same study concluded that adherence is a concern in North America.

### 1.2. Patient- and Family-Related Challenges

With regard to children, if the mother (or other caregiver) is infected, then she is struggling with her own illness, psychosocial factors, medication regimens, and most often financial burden due to expenses incurred on her own therapy, child's therapy, and associated cost of medical treatment [21, 22]. All of these produce negative influences on adherence. Empirical evidence increasingly suggest that user fees in some centers for antiretroviral therapy (ART) and HIV/AIDS care decrease adherence [23, 24]. These factors on top of the caregivers' and patients' experience, knowledge and beliefs on ART [25], reduce the caregiver's ability to provide proper care to the child, thereby affecting the necessary adherence over time [26–31]. Furthermore, factors such as age (especially infancy and adolescence have a negative effect) [19], refusal of treatment, knowledge of HIV status, clinical stage, and depressive symptoms, male gender, and changes in health status (improvement as well as deterioration) have also been identified as important factors which affect adherence to HAART (highly active antiretroviral treatment) in pediatric patients [22, 27–30, 32]. Denial and fear of HIV status, misinformation, and misconceptions about HIV (for instance beliefs that ART cures HIV [16]), low availability, accessibility, and acceptance of therapy are some of the obstacles among HIV-infected adolescents.

It is known that mothers tend to hide HIV infection status from their children and disclosure is often delayed until adolescence [33]. Reddi and colleagues show that only 7.9% children had been made aware of their own HIV infection status in their study in South Africa [34]. Disclosure of HIV infection status is a critical step and has obvious implications for adherence. Starting the disclosure process as early as 8-9 years of age and combining it with specific support, as suggested (http://www.hivatis.org) may result in increased adherence in children. There are similar reports that indicate lack of disclosure as predictors of poor adherence in adults [35]. Self-perceived family support and/or the family's and the household's knowledge of the patient's HIV infection status are considered important predictors of adherence [36].

### 1.3. Stigma- and Discrimination-Related Challenges

Stigma, on top of the general knowledge of the population about HIV/AIDS and ART treatment, is an important determinant of adherence in the settings of sub-Saharan countries according to studies conducted recently [17, 37–39].

Social or family stigmatization and fear of the consequences of revealing HIV infection status to sexual partners are closely related to poor adherence [40]. Family plays a crucial role in any kind of treatment in children [41] or adults [42]. Major issues related to family or caregiver that influence adherence include presence of anxiety; depression [37, 43, 44]; active substance abuse [37]; the presence of HIV infection in another family member; fear of disclosure of HIV positivity to the family; family disruptions; belonging to racial minorities or other vulnerable groups of the population.

Family and community members can both play a positive and negative roles in ART treatment initiation and adherence [42, 45]. For instance, the stigma associated with HIV infection or AIDS may be more severe than that of other illnesses, creating barriers to treatment initiation and support for adherence that might otherwise be available [42, 46]. On the positive side, family members and friends can play the role of treatment partners and provide much needed support [39, 42, 47].

Patients need to be encouraged by health care workers to disclose their status. However, studies of interventions to facilitate disclosure are lacking. Social institutions like the church, nongovernmental organizations (NGOs), and food aid services play a crucial role in issues ranging from creating awareness about the illness, mobilizing support, facilitating treatment, and promoting adherence [16, 42, 48]. For instance, in an evaluation program about the impact of family nutritional support during the first year of antiretroviral treatment in the west Africa region, family nutritional support for persons living with HIV initiating antiretroviral treatment showed a positive impact after six months [49].

### 1.4. Substance- Abuse-Related Challenges

Drug abuse and alcohol consumption are factors that further threaten proper adherences to ART. Studies have consistently shown that active alcohol use and substance abuse makes it more difficult for patients to adhere to treatment [50–53]. For instance, in Botswana nearly 40 percent of the patients surveyed admitted missing a dose because of alcohol consumption [46]. Similar studies also indicate that alcohol is highly related to reduced adherence [54]. A systematic review in 2009 found that HIV/AIDS patients that used alcohol are 50–60% more likely to adhere less to their prescribed medications [55].

### 1.5. Socioeconomic Challenges

The patterns of infection have been shown to vary globally depending on the social and economic conditions of the country affected, with poverty having a significant role as a social determinant of HIV/AIDS and the spread of the virus as well as access and adherence to ART treatment [42, 56].

Common reasons reported for missed doses include financial trouble [38, 57] that prevent caregivers of children or adult patients from collecting medication on time [42], distance barrier or lack of transportation facilities to the ART clinic [37, 46], vomiting of medication without redosing, incorrect dosing by a caregiver, missed clinic appointments and pharmacy collections, confusion between multiple caregivers, and self-discontinuation or refusal by children [34, 58, 59]. Furthermore, patients' beliefs that medications need to be taken with food leads them to avoid taking medications whenever food is unavailable, interfering with adherence [42, 60]. Sometimes patients are forced to choose between paying for transportation to the ART facility and using the money for food [42, 57, 61]. Studies in Uganda and Tanzania reported that transportation costs are considered serious obstacles to taking ART [62, 63]. This has implications not only for day-to-day adherence but also losses to follow up [64]. Determinants of ART adherence for HIV-infected persons in sub-Saharan Africa were examined with ethnographic research methods at HIV treatment sites in Jos, Nigeria, Dar es Salaam, Tanzania, and Mbarara, Uganda. The findings indicate that individuals taking ART routinely overcome economic obstacles to ART adherence through a number of deliberate strategies aimed at prioritizing adherence: borrowing and “begging” transport funds, making “impossible choices” to allocate resources in favor of treatment, and “doing without” [65].

### 1.6. Medication-Related Challenges

Good adherence (i.e., more than 95%) was associated with beliefs regarding the positive impact of the medications on participants' quality of life. Characteristics of the commercially available drug formulations such as taste, palatability, size of pills, availability of liquid formulations, and adverse effects (e.g., metabolic complications, lipodystrophy) can significantly affect adherence. Furthermore, the complicated regimen [66] to be followed, such as the need for daily administration, dietary restriction, drug interactions, frequency of dosing, dosage, and therefore pill burden or amount of liquid, also influence child's adherence to therapy [26, 28, 31, 32, 56]. The above-mentioned medication-related factors are crucial in determining children's adherence to ART. 

Chesney [27] reported that factors associated with nonadherence included housing instability and length of treatment with antiretroviral therapy. According to a report by Van Dyke et al. [67], the main reasons mentioned by patients for nonadherence were taste (16%) and child refusal (16%) for ritonavir, and taste (9%) and interference of medication schedule with lifestyle (10%) for nelfinavir [67]. Side effects are also usually associated with irregular medication intake or stopping medication altogether. 

### 1.7. Health-Care- and Systems-Related Challenges

Structural factors not directly related to patient or medications can also influence adherence. Some researchers have even contended that these could be the most important barriers to ART adherence in resource limited settings [5]. Limited availability and accessibility of antiretroviral medications and healthcare facilities for diagnosis and treatment of HIV/AIDs, out-of-pocket payments, high cost of ART and other health services, presence of healthcare providers experienced in ART provision, patient-nurse and other provider relationships, health care providers' beliefs, waiting time and opening hours [16, 42, 59, 68–70], availability of counseling services, and social, economic, or psychological support for people living in both developing as well as developed countries can influence adherence positively or negatively [28]. Ensuring the privacy of ART clinics and waiting areas need to be given special emphasis as authors of this paper and others documented [16, 42]. For instance, Skovdal and colleagues reported about patients who refused to leave consultation rooms citing to nurses Mr. so and so is outside [42].

Adherence support and clinic policies are also important predictors of adherence [37] as well as lack of adherence monitoring mechanisms [10]. A recent study from South Africa indicates that improving adherence is cost effective and helps to reduce health care costs especially those of hospital care [71].

### 1.8. Interventions to Improve Adherence

Continuous monitoring of both adherence and correlating it with clinical outcomes will create an interactive feedback mechanism that could lead to optimal clinical states and improved quality of life for patients. There are needs for further research and development in the area of ART adherence, adherence support, and patient behavior.

Diagnosing and treating health problems such as depression, reducing substance abuse, improving patient and provider relationship, counseling and enhancing family, and community support mechanisms are shown to improve adherence, as well as intervening on modifiable barriers to adherence before starting ART [72, 73]. A meta-analysis by Amico and colleagues indicated that adherence interventions may be efficacious when targeted at individuals who are identified or anticipated to have poor adherence [74].

The few investigations of interventions indicate that electronic reminders, pill organizers, medication-event monitoring systems (MEMS) to record dosing behavior, use of internet, educations services, use of phones [75], and so forth can also enhance adherence. However, most of these technologies have not had thorough scientific evaluation and their efficacy and cost effectiveness may not be as high as expectations [5, 72, 76]. Cell phone message reminders and web-based interventions require patient resources and literacy which could create obstacles to their applicability in sub-Saharan Africa. A recent systematic review published by the Cochrane Database of Systematic Reviews reached similar conclusions. It cited diverse methodological problems and issues of study quality, among others as problems underlying the scant evidence on adherence improvement interventions and called for standardized and methodologically rigorous trials of interventions to improve and measure adherence to antiretroviral treatment [77].

## 2. Discussion

**Figure 1:**
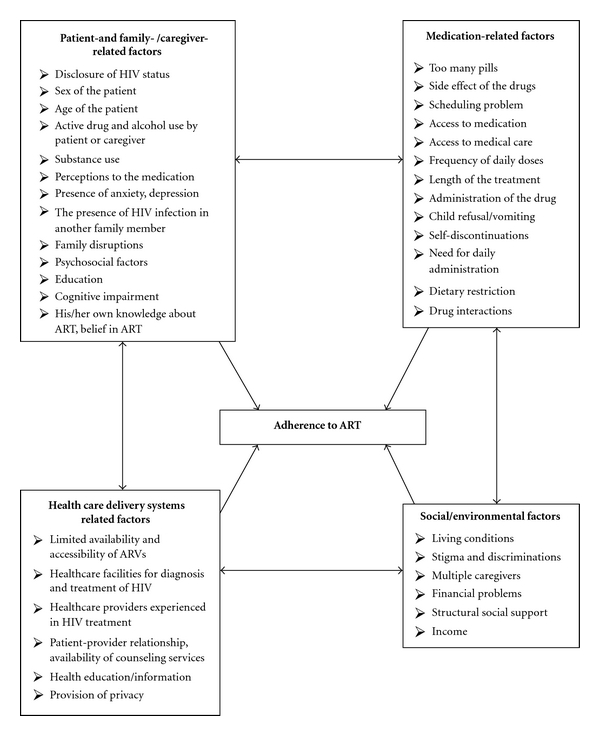
Identified factors for the challenges of adherence to ART.

African HIV/AIDS patients have similar or higher adherence levels compared to those of developed countries. The challenges of adherence to ART identified include factors related to patients and their families, socioeconomic factors, medication, and healthcare systems as summarized in Figure 1. This has implications for interventions to improve ART adherence and therefore the program needs to address these interrelated and multidimensional factors [78, 79]. In other words, ensuring adherence to treatment and retention requires an understanding of the multiple barriers that patients face and developing interventions that overcome these barriers. Long-term maintenance of adherence requires the integration of these interventions into sustainable programs that ensure a reliable supply of drugs, patient education, and ongoing support [80].

Low adherence to treatment has been associated with higher hospitalization rates, productivity loss, disease progression, and death in both high-income and resource-limited settings [35]. It is clear that adherence problems can constitute a significant barrier to ART programs in African countries or elsewhere. Without regulated access to ART, rapid emergence of drug-resistant viral strains and individual treatment failure is a potential threat and could curtail future treatment options and leading to the transmission of drug resistant strains of HIV [18]. We have identified that in order to increase adherence to the appropriate level there needs to be concerted efforts to evaluate and conduct operational research on ART service provision. These include use of new monitoring mechanisms, infrastructure, staffing, training of counselors, community support systems, and suitable drug formulations [78]. But currently there are several research gaps such as lack of capacity to survey the level of drug resistance in sub-Saharan Africa and testing of new tools for monitoring adherence.

This paper mainly focused on studies conducted on African HIV/AIDS patients. As a result, the predictors of ART adherence identified in the review may not necessarily be applicable to countries outside the region. Furthermore, currently there is no gold standard for measuring adherence. Because of this, most of the studies included used the most common forms of adherence assessment—patient recall and pill count—which have recognized biases. These include over reporting, recall, and social desirability bias [81–83]. We measured influential factors for short-term to medium-term adherence and that our conclusions on these factors may not necessarily be extrapolated to losses to follow up or retention to ART programs.

Health system barriers affect adherence, especially a regular and timely supply of medication to patients. An unreliable supply of medications can severely reduce patient adherence rates. In the majority of the sub-Sahara Africa countries they are manifested by weak procurement and supply management systems that lead to frequent shortages of ART and other essential inputs. In a survey of 91 low- and middle-income countries in 2008, 34% had experienced at least one stock out of a required ART medication [4].

In the future, it is possible that the encouraging trend of increased access to ART access may be further scaled up if governments and donors continue their commitment to the program. However, it is important that national governments take an increasing role in the program in order to make it sustainable. These include channeling of funds and policy commitments toward evaluation and improvement of the program. These also call for scale up of efforts to prevent the virus. In addition, policy measures to improve the socioeconomic status and empowerment of their citizens in general are very important.

## 3. Conclusion and Recommendations

There is a relatively modest level of adherence to antiretroviral treatment among HIV/AIDS patients in sub-Saharan Africa. However, it is challenged by a range of hierarchical and intricately related factors and there is substantial room for improvement of the ART programs in the region. Vulnerable groups such as children and adolescents need special attention by health workers and policy makers. There is also a need for adherence indicators and interventions that are applicable in the setting of developing countries.
